# Navigating the American Board of Surgery In-Training Examination (ABSITE) Success: Insights From Pre-assessment Practices in Preparing Surgical Residents for Competitive Sub-specialties

**DOI:** 10.7759/cureus.62896

**Published:** 2024-06-22

**Authors:** Armein Rahimpour, Missy Morrison, David A Denning, Paul Bown, Peter Ray, Rahman Barry

**Affiliations:** 1 General Surgery, Marshall University Joan C. Edwards School of Medicine, Huntington, USA; 2 Plastic Surgery, King’s Daughters Medical Center, Ashland, USA; 3 Plastic and Reconstructive Surgery, Marshall University Joan C. Edwards School of Medicine, Huntington, USA

**Keywords:** assessment tool, education in general surgical residency, surgical education, truelearn, absite

## Abstract

Introduction

The American Board of Surgery In-Training Examination (ABSITE) is a critical tool in assessing surgical residents' readiness for board certification and clinical practice. While various factors influencing ABSITE performance have been examined, the impact of innovative educational resources, such as TrueLearn, remains underexplored. TrueLearn's adaptive learning algorithms and comprehensive question banks offer a promising adjunct to traditional study methods. This study investigates the relationship between TrueLearn utilization and ABSITE performance among general surgery residents.

Methods

This retrospective study, ethically approved by the Marshall University Institutional Review Board (IRB No. 2097669-1), analyzed the performance of general surgery residents at Marshall University from 2014 to 2022. Data were collected on ABSITE scores. Additionally, quiz percentages (Quiz %) and scores from two mock exams (Exam 1 and Exam 2), all provided by the TrueLearn platform, were included in the analysis. Descriptive statistics summarized the sample characteristics. Linear mixed models were employed to examine the associations between TrueLearn engagement and ABSITE performance, accounting for the correlated nature of the data and addressing any missing data at random. Statistical analyses were conducted using the Statistical Analysis System (SAS, version 9.4; SAS Institute Inc., Cary, NC), with significance defined as a p-value < 0.05.

Results

The study cohort included 58 residents from 2016 to 2022. Linear mixed model analysis revealed significant positive correlations between TrueLearn Quiz %, Exam 1 scores, and Exam 2 scores with ABSITE performance. A 1% increase in Quiz % was associated with a 0.77-point rise in ABSITE scores (95% CI: 0.65, 0.89; p < 0.0001). For Exam 1, each point increase corresponded to a 6.36-point increase in ABSITE scores (95% CI: 5.01, 7.7; p < 0.0001), while Exam 2 scores showed a 3.8-point increase per point (95% CI: 2.74, 4.86; p < 0.0001).

Discussion and conclusion

Our findings underscore the significant impact of TrueLearn engagement on ABSITE performance, with higher quiz percentages and mock exam scores predictive of better ABSITE outcomes. This suggests that regular use of TrueLearn's educational resources enhances residents' knowledge and exam readiness. These results advocate for the integration of innovative educational tools such as TrueLearn into surgical training programs to optimize study strategies and improve exam performance. However, the study's retrospective design and single-institution focus limit the generalizability of the findings. Future research should explore these relationships in diverse settings and specialties and consider additional factors influencing ABSITE performance. This study highlights the positive association between TrueLearn utilization and ABSITE performance among general surgery residents, emphasizing the importance of innovative educational resources in surgical training. By enhancing engagement with platforms such as TrueLearn, surgical programs can improve residents' readiness for high-stakes examinations, ultimately contributing to the development of proficient surgical practitioners.

## Introduction

The American Board of Surgery In-Training Examination (ABSITE) stands as a cornerstone assessment tool in the arduous journey of surgical residency, offering invaluable insights into the readiness of aspiring surgeons for the rigors of clinical practice and board certification [1]. While numerous determinants of ABSITE performance have been scrutinized, ranging from standardized exam scores such as the United States Medical Licensing Examination (USMLE) to the volume of surgical cases encountered during residency [2,3], the influence of innovative educational resources, such as TrueLearn, remains an intriguing yet underexplored domain.

TrueLearn, with its adaptive learning algorithms and comprehensive question banks, has emerged as a potent adjunct to traditional study methods, promising significant enhancements in ABSITE scores and overall educational outcomes [4]. Despite its growing prominence, however, the precise impact of TrueLearn utilization on ABSITE performance among general surgery residents remains inadequately elucidated.

In the competitive milieu of surgical education and fellowship applications, where securing high ABSITE scores can be pivotal in shaping career trajectories and fellowship opportunities, unraveling the relationship between TrueLearn engagement and ABSITE outcomes assumes paramount importance. Our retrospective study endeavors to bridge this gap by delving into the intricate interplay between TrueLearn usage patterns, quiz participation, mock exam performance, and ABSITE scores among general surgery residents.

By leveraging longitudinal data spanning multiple cohorts of residents over several years, we seek to discern nuanced correlations and trends, shedding light on the effectiveness of TrueLearn as a tool in surgical education. In addition, we aim to unravel the intricate nuances of this relationship, offering actionable insights and evidence-based recommendations to optimize educational strategies and resources. Our study aspires to furnish empirical evidence to inform educational policy and practice, offering valuable guidance to surgical educators, program directors, and aspiring surgeons alike. By elucidating the role of TrueLearn in fostering ABSITE readiness and proficiency, we endeavor to contribute substantively to the ongoing discourse surrounding surgical education, training methodologies, and the cultivation of a competent, resilient, and diverse cadre of surgical practitioners.

## Materials and methods

The research protocol for this study was ethically approved by the Marshall University Institutional Review Board (IRB No. 2097669-1), granting authorization for data collection and analysis. The study focused on assessing the performance of general surgery residents enrolled at Marshall University during the period spanning 2014 to 2022. The inclusion criteria encompassed all residents working at the hospital, including both categorical and preliminary residents. No residents were excluded, as no exclusion criteria were applied. The study utilized data from TrueLearn, a comprehensive educational platform widely employed in medical training and preparation for standardized examinations. TrueLearn offers a range of practice resources, including quizzes and mock exams, designed to simulate the conditions of real assessments such as the ABSITE. These resources provide learners with valuable opportunities to reinforce their knowledge, identify areas for improvement, and gauge their readiness for high-stakes examinations. Comprehensive datasets were compiled, encompassing ABSITE scores, Quiz %, Exam 1, and Exam 2. The "Quiz %" represents the overall percentage of correct responses obtained in quizzes administered through the TrueLearn platform. Similarly, "Exam 1" and "Exam 2" denote the initial and subsequent mock examinations undertaken within the TrueLearn system, respectively. To conduct our investigation, we employed descriptive statistics to summarize the characteristics of our sample, with continuous variables presented as means ± standard deviations (SD) and categorical variables as numbers (N) and percentages (%). Data were collected longitudinally over multiple years, and linear mixed models were utilized to account for the correlated nature of the data and address any missing data at random. We visualized the relationship between each predictor (overall TrueLearn quiz percentage and 2 TrueLearn mock exams) and ABSITE performance by plotting observed values alongside fitted regression lines. All statistical analyses were performed using the Statistical Analysis System (SAS, version 9.4; SAS Institute Inc., Cary, NC), with statistical significance defined as a two-sided test with a p-value < 0.05.

## Results

Linear mixed models

Our analysis using linear mixed models revealed significant associations between quiz percentage, exam scores, and ABSITE performance among general surgery residents (Table 1). The analysis includes two outcome variables: ABSITE score and ABSITE %, with their respective predictors.

**Table 1:** Investigating the Influence of Quiz % and Exam Scores on ABSITE Performance Using Linear Mixed Models.

Predictors	ABSITE Score		ABSITE %	
	Beta (95% CI)	p-value	Beta (95% CI)	p-value
Quiz %	7.61 (6.49,8.73)	<0.0001	0.77 (0.65,0.89)	<0.0001
Exam 1	6.36 (5.01,7.7)	<0.0001	0.61 (0.47,0.75)	<0.0001
Exam 2	3.8 (2.74,4.86)	<0.0001	0.33 (0.22,0.43)	<0.0001

Quiz %

For the ABSITE score, the beta coefficient is 7.61 with a 95% confidence interval (CI) ranging from 6.49 to 8.73 and a p-value of < 0.0001. This indicates that, for every 1% increase in Quiz %, ABSITE scores increase by an average of 7.61 points. The 95% CI suggests that this increase is statistically significant and likely falls between 6.49 and 8.73 points. For the ABSITE %, the beta coefficient is 0.77 with a 95% CI of 0.65-0.89 and a p-value of < 0.0001. This means that, for every 1% increase in Quiz %, the ABSITE percentage increases by an average of 0.77 points. This result is also statistically significant, with the 95% CI indicating the increase is between 0.65 and 0.89 points.

Exam 1

For the ABSITE score, the beta coefficient is 6.36 with a 95% CI ranging from 5.01 to 7.7 and a p-value of < 0.0001. This shows that, for each one-point increase in the Exam 1 score, ABSITE scores increase by an average of 6.36 points. The 95% CI (5.01-7.7) confirms that this effect is statistically significant. For the ABSITE %, the beta coefficient is 0.61 with a 95% CI of 0.47-0.75 and a p-value of < 0.0001. This indicates that, for each one-point increase in the Exam 1 score, the ABSITE percentage increases by an average of 0.61 points. The effect is statistically significant, as indicated by the 95% CI (0.47-0.75).

Exam 2

For the ABSITE score, the beta coefficient is 3.8 with a 95% CI ranging from 2.74 to 4.86 and a p-value of < 0.0001. This means that, for each one-point increase in the Exam 2 score, ABSITE scores increase by an average of 3.8 points. This is statistically significant with a 95% CI range of 2.74-4.86 points. For the ABSITE %, the beta coefficient is 0.33 with a 95% CI of 0.22-0.43 and a p-value of < 0.0001. This shows that, for each one-point increase in the Exam 2 score, the ABSITE percentage increases by an average of 0.33 points. The increase is statistically significant, as evidenced by the 95% CI (0.22-0.43).

Fitted regression line

**Figure 1:**
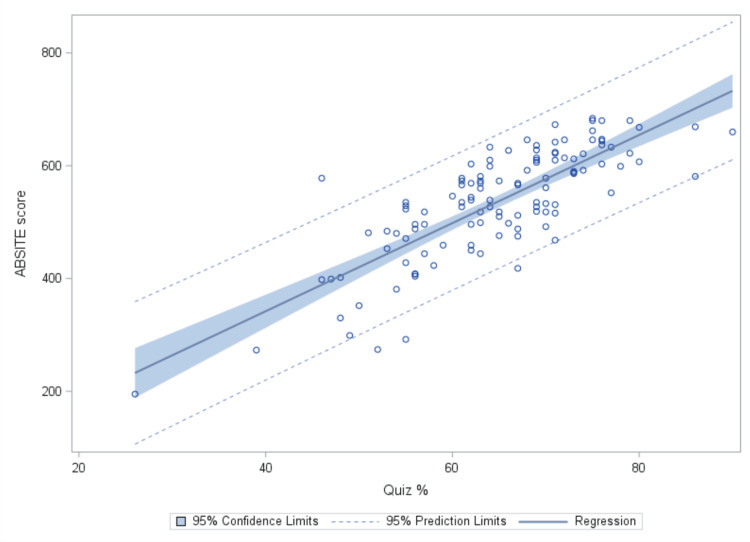
Observed Values and Fitted Regression Line for Quiz % vs. ABSITE Score.

To visualize the relationship between Quiz Percentage/Exam 1/Exam 2 and ABSITE score, we plotted the observed values alongside the fitted regression line. Figure 1 illustrates this relationship, with Quiz Percentage on the x-axis and ABSITE score on the y-axis. The scatter plot depicts the distribution of data points representing individual residents' quiz percentages and corresponding ABSITE scores. The fitted regression line, derived from the linear mixed model analysis, demonstrates the overall trend between Quiz Percentage and ABSITE score, indicating a positive correlation between the two variables.

**Figure 2:**
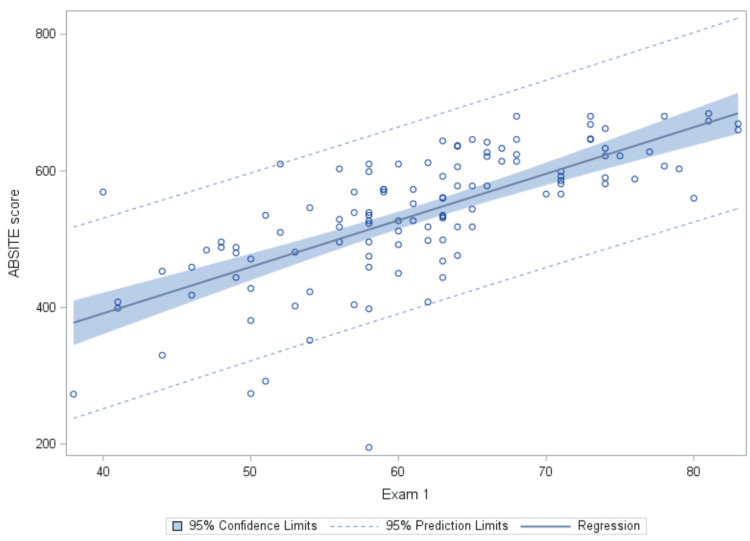
Observed Values and Fitted Regression Line for Exam 1 vs. ABSITE Score.

Figure 2 illustrates a similar relationship between Exam 1 on the X-axis and ABSITE score on the Y-axis. The scatter plot depicts the distribution of data points representing individual residents' quiz percentages and corresponding ABSITE scores. The fitted regression line, derived from the linear mixed model analysis, demonstrates the overall trend between Exam 1 and ABSITE score, indicating a positive correlation between the two variables.

**Figure 3:**
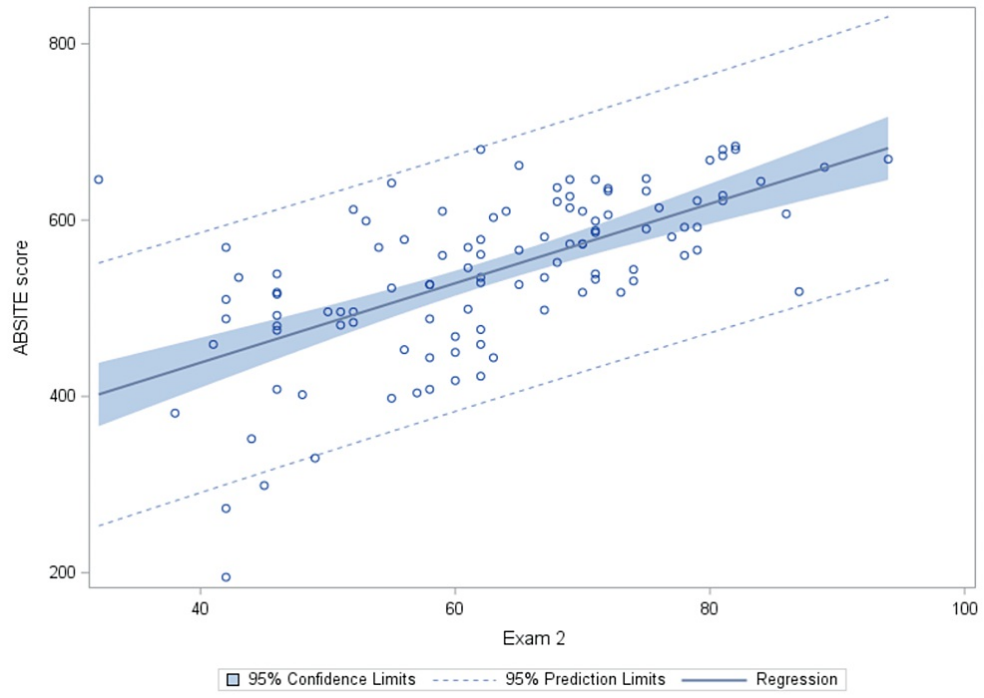
Observed Values and Fitted Regression Line for Exam 2 vs. ABSITE Score.

Figure 3 illustrates a similar relationship between Exam 2 on the X-axis and ABSITE score on the Y-axis. The scatter plot depicts the distribution of data points representing individual residents' quiz percentages and corresponding ABSITE scores. The fitted regression line, derived from the linear mixed model analysis, demonstrates the overall trend between Exam 2 and the ABSITE score, indicating a positive correlation between the two variables.

## Discussion

By exploring the significance of TrueLearn engagement, quiz participation, and mock exam performance in relation to ABSITE outcomes, we aim to provide valuable insights for educators, program directors, and surgical trainees alike.

Our study underscores the pivotal role of the ABSITE as a cornerstone assessment tool in surgical residency, offering invaluable insights into residents' readiness for board certification and clinical practice [5]. ABSITE scores of fifth-year general surgery residents have been identified as a reliable predictor of success on the General Surgery Qualifying Examination (GSQE), the first of two exams required for board certification in general surgery [5,6]. Besides its function as a diagnostic tool for evaluating individual performance, the ABSITE serves as a benchmark for evaluating the effectiveness of educational interventions and training methodologies. Nevertheless, it is notable that a recent study found that over 75% of participating programs reported ABSITE scores to be of low priority or not a priority [7]. Furthermore, success on the ABSITE has been shown in other studies to correlate with preliminary residents persisting in pursuing their career goals even after an unsuccessful first match, and it has allowed program directors to provide valuable career guidance [8].

In our investigation, we observed a significant association between TrueLearn engagement, quiz participation, and ABSITE performance among general surgery residents. Specifically, higher quiz percentages were predictive of better ABSITE scores, highlighting the utility of regular engagement with educational resources in bolstering residents' knowledge and readiness for high-stakes examinations. This finding aligns with previous research emphasizing the importance of active learning strategies and spaced repetition in optimizing educational outcomes [1,2].

Furthermore, our study elucidated the positive impact of mock exam performance on ABSITE outcomes, indicating that residents who performed well on TrueLearn mock exams tended to achieve higher ABSITE scores. This suggests that the practice and simulation provided by TrueLearn contribute to improved performance on the actual examination, underscoring the effectiveness of simulation-based learning methodologies in surgical education [3,4].

Our findings have far-reaching implications beyond individual performance, influencing educational policy and practice in the field. By highlighting the role of TrueLearn in enhancing ABSITE readiness and proficiency, our study underscores the importance of integrating innovative educational resources into surgical training curricula. Incorporating platforms such as TrueLearn can bolster the effectiveness of educational interventions, optimize study strategies, and ultimately elevate ABSITE performance among general surgery residents. However, it is noteworthy that the current utilization of question banks in educational preparation is limited. In a study examining 41 programs, only 50% reported using question banks as a preparation tool, with the Surgical Council on Resident Education (SCORE) being the predominant resource employed (89%) [7].

Various methodologies for predicting ABSITE scores have been explored in previous research [1,9]. The presence of fitted regression lines serves a dual purpose: it enables residents to assess their performance and potential score, thereby informing their preparation strategies. Residents who are performing well based on their potential score may choose to maintain their current study regimen or even take additional steps to further solidify their knowledge. Conversely, program directors can easily identify residents who may require additional support based on their performance relative to their potential score. This proactive approach allows for timely intervention and tailored assistance to ensure that all residents receive the necessary resources and guidance to excel in their preparation for the exam.

However, it is essential to acknowledge the limitations of our study. While our findings provide valuable insights into the relationship between TrueLearn utilization and ABSITE outcomes, our study is inherently observational and retrospective in nature, limiting our ability to establish causality. Prospective studies with randomized controlled trials could provide more definitive evidence. Additionally, our study focused solely on general surgery residents at Marshall University, which may limit the generalizability of our findings to other institutions or surgical specialties. Future research should replicate these findings across diverse settings and specialties. Thirdly, our study did not explore potential confounding variables or longitudinal trends comprehensively.

Further limitations of this study include the inclusion of both preliminary and categorical general surgery residents across all five years of residency. Future research could benefit from excluding preliminary residents and analyzing each class year separately. At Marshall University, TrueLearn is integrated into the educational package, alleviating the need for additional fees from residents and facilitating easier access. This practice could be implemented across programs to encourage resident participation.

Moving forward, future research endeavors should aim to replicate our findings in diverse settings and explore additional factors that may influence ABSITE performance, such as socioeconomic status, educational background, and residency program characteristics. Other factors such as the number of questions per quiz, mode of the quiz (tutor, timed, etc.), and number of quizzes taken can also be explored. Moreover, longitudinal studies tracking residents' progress over the course of their training could provide valuable insights into the long-term impact of TrueLearn engagement on surgical competency and clinical outcomes. Lastly, the development of evidence-based guidelines for integrating educational technologies such as TrueLearn into surgical curricula can enhance training programs globally. Addressing these avenues will further advance surgical education and training, ensuring the development of competent and skilled surgeons equipped for modern healthcare challenges.

In conclusion, our study contributes to the growing body of literature surrounding surgical education and assessment methodologies, offering valuable insights into the effectiveness of TrueLearn as a preparatory tool for the ABSITE. By elucidating the relationship between TrueLearn engagement, quiz participation, mock exam performance, and ABSITE outcomes, our research informs evidence-based interventions, educational initiatives, and policy reforms aimed at optimizing surgical training and enhancing the proficiency of future surgical practitioners. Through collaborative efforts and scholarly inquiry, we can continue to advance the field of surgical education, ensuring the development of competent, resilient, and diverse surgical leaders equipped to meet the evolving challenges of modern healthcare delivery.

## Conclusions

In conclusion, our study underscores the significant association between TrueLearn engagement and ABSITE performance among general surgery residents. By leveraging longitudinal data and employing robust statistical analyses, we have provided compelling evidence of the positive impact of TrueLearn utilization on residents' readiness and proficiency in surgical knowledge assessment. These findings underscore the pivotal role of innovative educational resources in enhancing surgical education and training, offering valuable insights to educators, program directors, and aspiring surgeons alike. Moving forward, prioritizing evidence-based educational interventions and fostering a culture of continuous learning and improvement can further optimize the educational outcomes of surgical trainees, ultimately contributing to the cultivation of competent and proficient surgeons prepared to meet the evolving demands of contemporary healthcare.
